# Association between Slip Severity and Muscle Synergies of Slipping

**DOI:** 10.3389/fnhum.2017.00536

**Published:** 2017-11-07

**Authors:** Mohammad Moein Nazifi, Kurt E. Beschorner, Pilwon Hur

**Affiliations:** ^1^Human Rehabilitation Group, Department of Mechanical Engineering, Texas A&M University, College Station, TX, United States; ^2^Department of Bioengineering, University of Pittsburgh, Pittsburgh, PA, United States; ^3^Department of Industrial Engineering, University of Wisconsin-Milwaukee, Milwaukee, WI, United States

**Keywords:** motor control, muscle synergy, slip, gait, fall

## Abstract

Falls impose significant negative impacts to the US population and economy. A significant number of falls may be prevented via appropriate slip-responses since a strong relation exists between slips and falls. More importantly, as severe slips are more prone to result in a fall, identifying severe slippers along with the responsible factors for their adverse motor control and severe slipping should be the highest priority in fall prevention process. Previous studies have suggested that muscle synergies may be building blocks of the central nervous system in controlling motor tasks. Muscle synergies observed during slipping (‘post-slip-initiation synergies’ or ‘just briefly,’ ‘slipping muscle synergies’), may represent the fundamental blocks of the neural control during slipping. Hence, studying the differences in slipping muscle synergies of mild and severe slippers can potentially reveal the differences in their neural control and subsequently, indicate the responsible factors for the adverse post-slip response in severe slippers. Even though the slipping muscle synergies have been investigated before, it still remains unclear on how the slip severity is associated with the slipping muscle synergies. More importantly, muscle synergies can be interpreted not only as neural blocks but also as physical sub-tasks of the main motor task. Hence, studying the differences of slipping synergies of mild and severe slippers would reveal the discrepancies in sub-tasks of their post-slip response. These discrepancies help pinpoint the malfunctioning sub-function associated with inadequate motor response seen in severe slippers. Twenty healthy subjects were recruited and underwent an unexpected slip (to extract their slipping synergies). Subjects were classified into mild and severe slippers based on their Peak Heel Speed. An independent *t*-test revealed several significant inter-group differences for muscle synergies of mild and severe slippers indicating differences in their neural control of slipping. A forward dynamic simulation was utilized to reveal the functionality of each synergy. Decomposition of slipping into sub-tasks (synergies), and finding the malfunctioning sub-task in severe slippers is important as it results in a novel targeted motor-rehabilitation technique that only aims to re-establish the impaired sub-task responsible for the adverse motor-response in severe slippers.

## Introduction

In 2014, the death toll due to slips, trips, and falls ranked second among occupational injuries (Bureau of Labor Statistics US Department of Labor, 2015a). Falls account for about a quarter of total days-away-from-work cases in the US (Bureau of Labor Statistics US Department of Labor, 2015b). More troubling, fall-related injuries have been showing a growing trend recently (Bureau of Labor Statistics US Department of Labor, 2015a). Considering numerous impacts of falls on public health and the economy (Morrison et al., 2013; Bureau of Labor Statistics US Department of Labor, 2015b; Honeycutt et al., 2016), research that describes slipping and the recovery process is of great importance.

Slipping is one of the main triggers to falling (Gao and Abeysekera, 2004; Di Pilla, 2009). Considering the relation between slips and falls, preventing slips plays a key role in fall prevention. Nevertheless, not all slips result in a fall. “Severe slips” (sometimes referred to as hazardous slips) are more likely to result in falls compared to “mild slips.” To assess the severity of a slip, several studies have tried to introduce different measures such as PHS or slipping distance as the key factors in assessing slip severity (Perkins, 1978; Strandberg and Lanshammar, 1981; Lockhart et al., 2003a). Lockhart et al. (2003a) claimed that slipping distances and speeds higher than 3.91 cm and 1.44 m/s should be considered severe in younger adults. Interestingly, being a “severe slipper” can be considered as a characteristic of an individual (Lockhart et al., 2003a). Lockhart et al. (2003b) found that even though younger and older adults have the same potential for slip initiation, older adults slipped more severely compared to younger adults. This fact indicates that severe slipping is also highly related to the post-slip-initiation motor responses rather than motor behaviors before the slip initiation. Consequently, identification of severe slippers and prevention of severe slips may have significant potential for targeting interventions and preventing falls.

*Muscle synergy* hypothesis suggests that the CNS may control motor tasks using a small set of co-activated muscles, or muscle synergies (d’Avella and Bizzi, 2005; Ting and Macpherson, 2005). Each set of these grouped co-active muscles, that form a muscle synergy, can be recruited with an independent activation signal, or *activation coefficient*. Also, several studies suggested that each muscle synergy may represent a sub-task of the original motor task (d’Avella et al., 2003; Neptune et al., 2009). Previous studies have discussed the beneficial aspects of a muscle synergy approach in studying motor tasks, like walking and slipping (Nazifi et al., 2017). A muscle synergy approach highly facilitates analysis of the coordination of the interlimb muscles, since the muscle synergy hypothesis claims that all muscles with the same neurological origin that are activated together appear in the same synergy. However, traditional EMG analysis fails to decompose co-activated muscles into the same control block (synergy) (Chambers and Cham, 2007; Qu et al., 2012). Moreover, another main advantage of the muscle synergy approach is that it would help identify the sub-tasks of the original motor-task. Not only would these sub-tasks facilitate diagnosis of the severe slippers, but also they might result in designing of a targeted motor rehabilitation based on the impaired sub-tasks (Allen et al., 2013; Roh et al., 2013).

Although previous studies have extracted and studied slipping response muscle synergies in young adults (Nazifi et al., 2016, 2017), no study tried to relate slipping muscle synergies to slip severity. In this sense, this study proposes the first step to investigate the cause of severe slips or discrepancies between the interlimb coordination of the mild slippers compared to severe slippers while experiencing a slip. The objective of this study is to compare the slipping muscle synergies and activation coefficients of “severe slippers” and “mild slippers” to quantify differences in coordination between the two groups. Such differences in muscle synergies, if found, can potentially be related to severity index of an individual. Also, the function of each synergy would be investigated to reveal the sub-function of each synergy during slipping. We hypothesize that the slipping muscle synergies would differ between mild slippers and severe slippers, indicating the malfunctioning synergies responsible for the adverse slip response of the severe slippers. Also, as the previous studies have revealed similarities between the control of the gait and slipping (Nazifi et al., 2017) we hypothesize that the physical sub-functions of some synergies (after being revealed via forward simulation) would be common with known sub-functions of the gait.

## Materials and Methods

### Subjects

A total number of 20 young adults [9 females and 11 males, age (mean ± SD): 23.6 ± 2.52] were recruited for this study. Subjects were excluded in case of a history of neurological, orthopedic, cardiovascular, pulmonary, and gait abnormalities. The experiment took place upon approval of Institutional Review Board at the University of Pittsburgh. The deidentified dataset was then transferred to Texas A&M University for further analysis with approval from IRB of both Universities. All subjects gave written consent before their participation.

### Measurements, Experimental Protocol, and Data Processing

**FIGURE 1:**
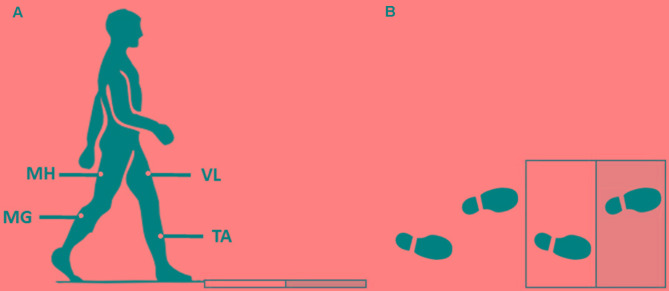
The side **(A)** and top view **(B)** of the experimental setup for the walkway and force plates. Gray surface indicates the slippery (contaminated) force plate in slip trials.

Participants were asked to walk in a pathway at their self-selected speed. There were two force plates embedded in the pathway. To have each of the force plates receive exactly one foot-strike (the right foot first, and then the left foot second), the starting location of each subject was adjusted (**Figure 1**). To induce an unexpected slip, subjects were assured that the surface would be dry during trials. However, after two to three normal walking trials, the surface of the second force plate was contaminated by applying a solution (75% glycerol, 25% water). To minimize the inter-subject variation of friction force, all subjects wore the same brand/model of polyvinyl chloride hard-soled shoes that matched their sizes. The lights were dimmed throughout the experiment to minimize the visual clues about the slippery surface. Also, to catch the subjects in case of a total loss of balance after experiencing a slip, a safety harness was provided.

EMG data were recorded at 1080 Hz to extract the muscle synergies. Surface EMG electrodes were used to record the activation of four major leg muscles according to Chambers and Cham (2007): *MH* (i.e., the primary knee flexor/hip extensor), *TA* (the main ankle dorsiflexor), vastus lateralis (*VL*) (hip flexor/knee extensor), and *MG* (knee flexor/ankle plantarflexor). The data were recorded from both right/trailing/non-slipping leg and left/leading/slipping leg. Joint kinematics and PHS was captured using a motion capture system (Vicon 612, Oxford, United Kingdom) at 120 Hz. Also, kinetic data and ground reaction forces were collected at 1080 Hz using the force plates.

The EMG data were demeaned, rectified, filtered (4th order low-pass Butterworth filter, cut-off: 15 Hz), normalized (to the maximum activation recorded for each muscle of every individual), and integrated for every 10 ms of the activity (d’Avella et al., 2003). Previous studies have suggested that the aforementioned four muscles have an activation onset time of less than 175 ms in response to an unexpected slip (Cham and Redfern, 2002; Marigold et al., 2003; Hur and Beschorner, 2012; Nazifi et al., 2017). Hence, the first 300 ms after the slip initiation (i.e., the heel strike moment) was used in slipping muscle (i.e., the synergies observed while the subjects were experiencing a slip or ‘post-slip-initiation’ muscle synergies) synergy extraction. Using an iterative non-negative matrix factorization (MATLAB 2014a, Mathworks, Natick, MA, United States) consistent with previous research (Ting and Macpherson, 2005; Clark et al., 2010; Roh et al., 2013; Nazifi et al., 2017) the processed EMG data (M) was decomposed into slipping synergies (W) and activation coefficients (C) (Eq. 1): first, matrices W and C were initialized with random values (positive). Then, using MATLAB function *fmincon*, the W matrix was updated. C matrix was then calculated to minimize the error (e). This procedure repeated until meeting the desired error bounds. Since previous research (Nazifi et al., 2017) has shown that four synergies are enough to reconstruct slipping data with a VAF > %95 (Eq. 2, note that F stands for Frobenius norm), in this study four slipping synergy were extracted and sorted using a reference subject that had the most similar behavior to all other individuals (Nazifi et al., 2017). Then, using the markers’ data (that includes the 3-D position of the heel), the instantaneous heel velocity was calculated. Then, using the PHS criterion, the subjects were classified into the mild and the severe slippers. Slips with a PHS of 1.44 m/s or greater were considered “severe,” and the rest were counted as “mild” (Lockhart et al., 2003b). Once the subjects were separated into severity sub-groups, the synergies of each group were reordered and sorted according to their similarity to each other (similarity was assessed via correlation coefficient, *r*) (Nazifi et al., 2017). To detect significant inter-group differences, an independent *t*-test (α = 0.05) was used for each of the muscle synergies and every time point of the activation coefficients using SPSS (v21, IBM, Chicago, IL, United States).

$$M_{30\times 8_{processed}}=\overset{n}{\underset{i=1}{\Sigma }}c_{i}w_{i}+e=C_{30\times n}\times W_{n\times 8}+e\,\;\left(C,W,e\geq 0\right)$$

$$VAF=1-\frac{||M_{30\times 8,\;processed}-{M_{30\times 8,\;rebuilt}||}_{F}^{2}}{{\left||M_{30\times 8,\;processed}|\right|}_{F}^{2}}$$

Lastly, to reveal the role of each synergy, OpenSim (SimTK, Stanford, CA, United States) was used to perform a forward simulation of each synergy. The activations resulting from each muscle synergy was separately fed to a generic model to observe the resulting joint torques. Using the provided generic 10 degree-of-freedom gait model in OpenSim, the model was first scaled to match to the anthropometric parameters of the reference subject (weight: 52.5 kg, height: 1.64 m). Reference subject presented the most similar behavior to all other subjects. According to Nazifi et al. (2017), the reference subject was chosen as the subject who had the largest correlation coefficient values (*r*) for any possible pair of synergies between the reference subject and all other subjects. Then, the 300 ms time course data of muscle activities resulting from each individual synergy were fed to the corresponding muscles in OpenSim while holding the lower limb joints in a static position (i.e., the same posture at the heel contact of the slipping limb). Finally, the resulting joint moments were studied.

## Results

**FIGURE 2:**
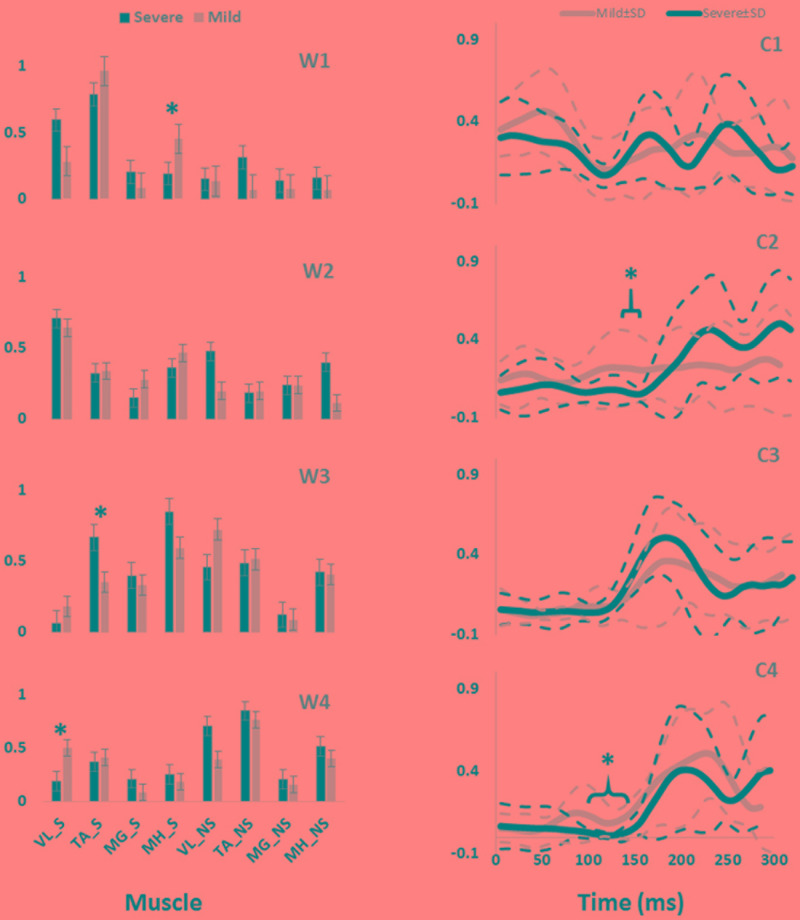
The slipping muscle synergies (W’s) and the corresponding activation coefficients (C’s) for both severity groups. Solid lines show the average value while dashed line shows one standard deviation. Asterisk indicate significant inter-group difference.

Four muscle synergies and their corresponding activation coefficients were extracted from the processed data according to our previous study (Nazifi et al., 2017). Based on PHS, 12 subjects were classified as mild slippers (PHS < 1.44 m/s) while the other eight were severe slippers (PHS ≥ 1.44 m/s). There was no difference observed in age, height, and mass of the severe slippers versus mild slippers (Information on each group is provided in **Table 1**). The averaged synergies and activation coefficients for each group are provided in **Figure 2**.

**Table 1:** Information of each severity group.

Mean (SD)	PHS (m/s)	Age	Mass (Kg)	Height (cm)	Sex (M/F)
Mild	0.63 (0.25)	24.17 (2.79)	68.41 (11.89)	171.75 (8.59)	5/7
Severe	1.87 (0.27)	22.75 (1.48)	70.00 (11.37)	175.19 (7.57)	6/2


Independent *t*-test detected several inter-group differences. Higher activation of *MH_S* in the first synergy (W1) was significantly different between mild and severe slippers (**Table 2**). Higher *MH_S* activation was associated with mild slips (**Figure 2**). Also, activation of the *VL_S* in the fourth slipping synergy (W4) was found to be different between mild and severe slippers (**Table 2**). Mild slippers showed a higher contribution of *VL_S* during their slips (**Figure 2**). Lastly, activation of the *TA_S* was different in the third muscle synergy (W3) (**Table 2**). Higher activation of *TA_S* was associated with severe slips (**Figure 2**).

**Table 2:** Variables that showed statistically significant differences between groups.

Variable		Mild	Severe	*p*-Value
W1	MH_S	0.45 (0.29)	0.19 (0.19)	0.040
W3	TA_S	0.35 (0.31)	0.67 (0.28)	0.032
W4	VL_S	0.50 (0.29)	0.19 (0.20)	0.017
C2	130*^ms^*–140*^ms^*	0.22 (0.24)	0.06 (0.07)	0.045
	140*^ms^*–150*^ms^*	0.12 (0.12)	0.01 (0.02)	0.010
C4	100*^ms^*–110*^ms^*	0.14 (0.16)	0.02 (0.03)	0.026
	110*^ms^*–120*^ms^*	0.12 (0.12)	0.01 (0.02)	0.010
	120*^ms^*–130*^ms^*	0.09 (0.09)	0.01 (0.02)	0.012
	130*^ms^*–140*^ms^*	0.09 (0.09)	0.02 (0.04)	0.043


**FIGURE 3:**
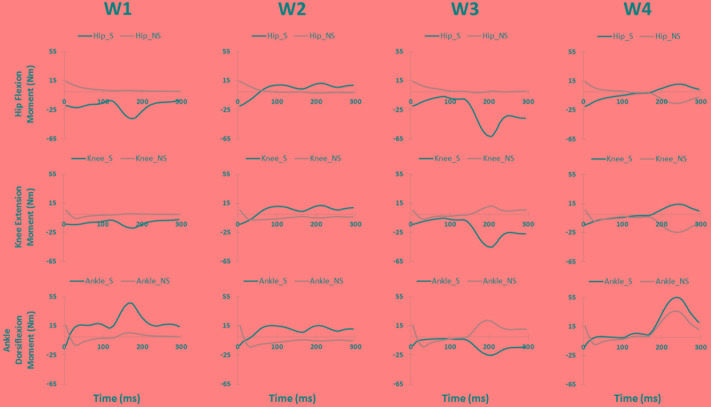
The joint moments (presented in rows) of the slipping (S) and non-slipping (NS) limb calculate via simulation for all slipping synergies (presented in columns).

Significant differences were also observed in the activation coefficient of two synergies. Mild slippers had significantly higher activations for the second synergy (C2) (**Table 2**) from 130 to 150 ms after the slip initiation (**Figure 2**). Additionally, mild slippers had higher activations for the fourth synergy (C4) from 100 ms until 140 ms after the heel strike on the slippery surface (**Figure 2**). These differences may indicate that the mild slippers activated their corresponding muscle synergies faster (earlier by 30–50 ms) than their severe slipper counter parts in response to a slip. The simulation results revealed the role of each muscle synergy during slipping (**Figure 3**). The first slipping synergy caused a significant hip extension, knee flexion, and dorsiflexion moment on the slipping limb (**Figure 3**). The second synergy mainly prompted hip flexion and knee extension moment on the slipping limb (**Figure 3**). The third muscle synergy resulted in a considerable hip extension, knee flexion, and ankle plantar flexion moment in the slipping limb as well as knee extension moment on the non-slipping limb. However, the fourth muscle synergy induced a substantial ankle dorsiflexion moment on the unperturbed limb. It also caused a distinct hip extension and knee flexion on the unperturbed limb.

## Discussion

The significant inter-group differences for muscle contributions and their activation coefficients show important aspects of post-slip-initiation responses. The similarities between the general trend of muscle contribution ratios in the synergies of different severity groups suggest that mild slippers and severe slipper use the same strategies in response to a slip. However, the differences observed in the activation coefficients may indicate that mild slippers can respond in a faster (**Figure 2**) or stronger (**Figure 2**) way, as opposed to having an overall stronger response throughout the slip. The first muscle synergy may be responsible for hip extension, knee flexion, and dorsiflexion of the perturbed limb (**Figure 3**). This synergy is likely to be responsible for the terminal swing phase of the gait. Moreover, a higher activation of the *MH_S* was observed in mild slippers in this synergy (*p*-value = 0.04) (**Figure 2**). Considering the role of the *MH* muscle in deceleration of the limb, it is suggested that the mild slippers can generate a greater deceleration at the terminal swing phase. The role of the second synergy in generating hip flexion and knee extension (**Figure 3**) matches with the secondary response to slips (Cham and Redfern, 2001) in which the mild slippers had a higher activation (**Figure 2**). This indicates that there is an association between severity mitigation and stronger activation of the secondary response to slips. The function of the third muscle synergy is likely to be hip extension, knee flexion, and ankle plantarflexion (**Figure 3**). This is a known sub-task during slipping, namely, the primary response to a slip (Cham and Redfern, 2001). However, excessive activation of the *TA_S* muscle was observed among severe slippers (*p*-value = 0.03, **Figure 2**), resulting in an excessive FFA and thus severe slips (Moyer et al., 2006). Lastly, the fourth muscle synergy caused significant effects on the unperturbed limb. It induced a distinct hip extension and dorsiflexion (**Figure 3**). This sub-function may be interpreted as another strategy to counter the slips called the “toe-touch.” Toe-touch is commonly practiced as an effective way to increase the base of support while slipping (Marigold et al., 2003). Moreover, the mild slippers showed faster activation for their toe-touch synergy (**Figure 2**). This may suggest that faster recruitment of toe-touch strategy is associated with less severity.

The results found in this research stay consistent with previous studies. Nazifi et al. (2017) claimed that a muscle synergy is strongly shared between walking and slipping. The aforementioned muscle synergy has very similar muscle contribution patterns to the first slipping synergy found in this study substantiating our claim about this synergy to be the shared muscle synergy between walking and slipping (so called, “deceleration synergy”). Additionally, Cham and Redfern (2001) claimed that the response to an unexpected slip can be decomposed into two fundamental components, namely, the primary and the secondary response. The primary response is responsible for bringing the slipping limb back near the center of mass while the secondary response tends to extend the slipping limb to maintain forward weight progression by shifting the center of mass over the base of support. The observed muscle activations and simulation results verify the concluded functionality of these muscle synergies in response to a slip. Finally, Marigold and Patla (2002) and Marigold et al. (2003) found the toe-touch response as a principal strategy required to maintain balance after a slip. Furthermore, he claimed that higher fall incidences in the elderly may be due to their inability in generating a fast toe-touch response, which further substantiates our finding about the faster activation pattern associated with mild slippers in the fourth muscle synergy.

Our conclusion about the first slipping synergy belonging to the terminal swing phase of the gait cycle and being the “deceleration synergy” comes from several observations: first, this muscle synergy (W1, **Figure 2**) has a dominant activation of *TA, VL*, and *MH* of the slipping limb. According to Rose and Gamble (2005), these muscles are activated during the final stage of the swing phase of the gait cycle. An eccentric (while lengthening) contraction of the *MH* (due to the activation of its antagonist, *VL*) should result in a smooth and effective deceleration of the swing limb. Also, the tibialis group will undergo an eccentric contraction to coordinate landing of the foot on the floor that verifies this interpretation. Hence, the observation of these muscles contributions in the first slipping synergy would result in the same physical sub-function as the deceleration of the limb in the terminal swing phase. Second, the activation patterns of the first slipping synergy also stay consistent with our suggested conclusion that the first slipping synergy is the “limb decelerator synergy,” because there is a considerable activation, compared to other muscle synergies in **Figure 2**, immediately after the heel contact (0–100 ms). This immediate activation after the heel contact proves that this muscle synergy is decelerating the limb in the terminal swing phase of the gait. That is because the muscular corrective responses to a slip happen 120–170 ms after the heel strike rather than immediately after heel strike (Cham and Redfern, 2001; Chambers and Cham, 2007; Hur and Beschorner, 2012). Consequently, observation of this significant activation between 0 and 100 ms post-heel strike indicates that this synergy is active even before the corrective response to a slip begin, and hence, belongs to the teminal phase of the gait cycle. The kinetics induced by this synergy (hip extension, knee flexion, ankle dorsiflexion) form the simulation study verifies the suggested functionality of this synergy in terminal swing phase and throughout slipping (**Figure 3**). Importantly, mild slippers showed a significantly higher activation of the *MH* muscle, which play the key role in decelerating the limbs in their terminal swing phase (Medved, 2000; Rose and Gamble, 2005; Lockhart and Kim, 2006). Hence, the association found between activation of hamstring muscle group and the mitigation of the slip severity suggests that the mild slippers possess a higher contribution of the “limb decelerator muscle” (hamstring) in their “limb decelerator synergy” (first slipping muscle synergy) prior to slip initiation. Also, another study suggested that higher knee flexion moment leads to more deceleration of the heel and reduced the risk of severe slips (Beschorner and Cham, 2008). This higher contribution indicates the higher capacity of mild slippers in slowing down their base of support and their slipping limbs right before the heel strike.

In the second synergy, the dominant activation of the *VL_S* resulted in significant hip flexion, knee extension, and plantarflexion (**Figure 3**, compare with other synergies). As *VL* plays an important role in supporting body weight, having a high activation level for *VL_S* suggests that the expected role of this synergy is the weight support on the slipping limb (Nazifi et al., 2017). This subfunction, known as the secondary response to a slip (Cham and Redfern, 2001), is crucial in slip responses since it can be considered as an attempt to continue gait and the forward weight progression on the slipping limb. This weight transfer to the slipping limb helps prevent knee buckling on the unperturbed limb. While there was no significant difference observed in the second muscle synergy (W2), the mild group showed a significantly higher level of activation for this muscle synergy (C2, **Figure 2**) between 130 and 150 ms after the heel strike. Considering the role of this synergy in the forward weight transfer, having a higher activation level offers a stronger weight support provided by the mild slippers compared to severe slippers. This results in a more effective weight transfer of the center of mass over base of support (Chambers and Cham, 2007). Failing to provide enough activation on *VL* of the slipping limb has been reported to be an involving factor in slip severity in other studies as well (Cham and Redfern, 2001; Chambers and Cham, 2007). The timing of the peak of activation in this synergy (about 200 ms post-heel strike, **Figure 2**) stays consistent with our speculation about its sub-task (i.e., the secondary slip response). The simulation results further verified the proposed sub-task for this synergy.

**FIGURE 4:**
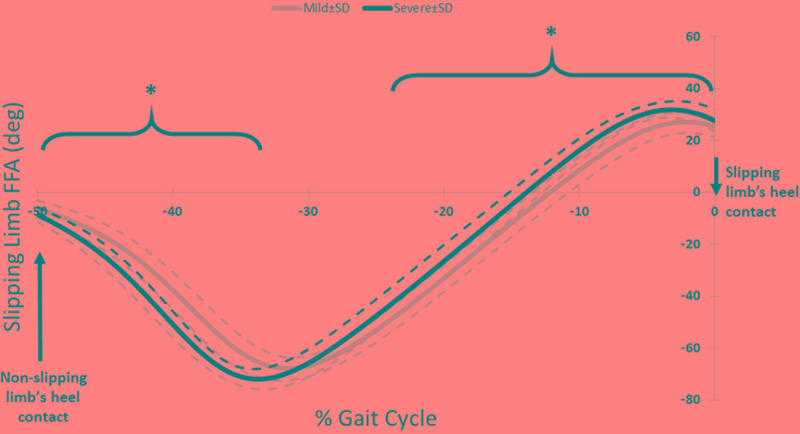
The FFA for the slipping limb for right before the heel strike of the slipping limb. Note that the slipping heel strike happens at 0%. The asterisks indicate significant differences while dashed lines represent one standard deviation.

The third muscle synergy was assumed to generate the known “primary slip response” (Cham and Redfern, 2001). This assumption was made based on the following reasons: first and foremost, the primary response tries to retrieve the slipping limb under the body which is achieved by the exertion of a knee flexion and hip extension moment (Cham and Redfern, 2001). The simulation indicates the same moments on the slipping limb (**Figure 3**), supporting the suggested function. Secondly, the timing of the peak activation of this synergy can provide further evidence about the proposed function. The activation becomes distinct around 160 ms after the heel strike on the slippery surface (C3, **Figure 2**). According to Cham and Redfern (2001), the active corrective responses becomes distinct about 150–200 ms after the perturbation; hence substantiating the proposed mechanical goal for the third muscle synergy. In other words, the peak of activation for primary response happens after the “terminal swing synergy” (W1) and before the “secondary response” (W2) (refer to **Figure 2**). On the other hand, higher activation of *TA_S* was observed in third slipping muscle synergy for severe slippers (W3, **Figure 2**) (*p*-value = 0.03). Pretibial muscles are highly activated during the early stance and terminal swing phase (Medved, 2000; Rose and Gamble, 2005). However, an excessive activation of *TA* muscle on the slipping limb is associated with severe slipping due to an excessive dorsiflexion of the foot. This finding can be also approached by point of view of the FFA. Moyer et al. (2006) claimed that severe slippers had a significantly higher FFA compared to their mild slipper counterparts at the heel strike moment. To quantify the FFA in our experiment, the markers data were used to study the angle of the slipping limb right before the heel strike (**Figure 4**). The calculated angles for both mild and severe slippers were examined for inter-group differences using an independent *t*-test (SPSS v21, IBM, Chicago, IL, United States). Interestingly, the results verified that the severe slippers had a higher FFA prior to their heel contact (*p-*value < 0.05) (**Figure 4**). Although unlike Moyer’s study, our experiment has mainly focused on investigating the post-slip-initiation incidents rather than pre-slip parameter, there is a high possibility that the association of the high *TA_S* activation with severe slips stays in the same line with Moyer’s claim about the higher FFA in severe slippers.

In the fourth muscle synergy, the most activated muscles belong to the non-slipping limb. Considering the dominant activations (*TA, MH, VL*), the function of the fourth synergy is to prevent trips and generate a toe-touch response for the non-slipping limb. The trip avoidance happens due to the high activation of *TA_NS* (Nazifi et al., 2017) (also supported with the simulation results, compare ankle moments in **Figure 3**), while the toe-touch is achieved by flexing the hip (**Figure 3**). Toe-touch is commonly practiced as an effective way to increase the base of support while slipping via extension of the unperturbed limb to touch the ground. On the other hand, a significant higher activation of *VL_S* (*p*-value = 0.017) in the “toe-touch” synergy for mild slippers (W4, **Figure 2**) suggests that the slipping limb supports the body weight when the trailing limb has not yet touched the ground to provide any weight support. One interpretation could be that the severe slippers were unable to maintain their weight support on the slipping limb to secure enough time for the toe-touch to happen and increase their stability. Moreover, the activation pattern for the fourth synergy (C4, **Figure 2**) was also significantly different between different severity groups. Mild slippers were able to recruit their “toe-touch” synergy faster than severe slippers (**Figure 2**). This result suggests that not only were mild slippers able to provide a better weight support (*VL_S* activation), but also they could execute the toe-touch strategy faster. This interpretation stays consistent with currently existing literature suggesting that a slow toe-touch in elderly is responsible for more frequent fall incidents (Marigold et al., 2003).

The findings of this paper may facilitate development of a synergy-based targeted motor rehabilitation, which may be a highly convenient and effective rehabilitation method. Targeted motor rehabilitation tries to design interventions that only stimulates and rehabilitates the impaired sub-function of a given motor-task to re-establish the sub-tasks and improve the overall motor-skill. This technique has already been proven to be beneficial in improving motor skills in patients (Dipietro et al., 2007). Subsequently, our findings about the sub-optimally performing sub-functions in severe slippers could be used in developing novel interventions that only stimulates the lost or impaired sub-tasks of slipping in order to transform severe slippers to mild slippers. Future studies will assess the extent of improvements in severity index of severe slippers after exposure to the aforementioned training method.

There were also a few limitations associated with this study. First, although this study revealed the association between severe slipping and adverse post-slip-initiation response, it is still unclear if this relation is causal or not. More investigations are required to clarify if there is a causal relation between slip severity and adverse post-slip-initiation response. To resolve this limitation, in future studies we will use interventions to improve the slip-response in subjects to see if it results in mitigation of severity. We believe that ‘severe slipping synergies’ will evolve to ‘mild slipping synergies’ as the subjects undergo slip trainings (Alnajjar et al., 2013). Also, a correlation analysis would further clarify the relation between slipping muscle synergies and different slip severities and result in a relation the severity index to the level of deviations observed from the synergies of reference mild slippers (Cheung et al., 2012). Lastly, model-based experiments can be performed to easily modulate experimental conditions and examine the causal relationship. In future works a wider range of age would be considered to recruit older adults. Also, future studies can perform kinematic analysis (only kinetic analysis was used in this study) in order to further investigate the functionality and importance of each muscle synergy of slipping.

## Conclusion

This study has investigated the inter-group differences in slipping muscle synergies of the mild and severe slippers and identified several significant differences. This study also utilized a forward dynamic simulation in order to study the sub-task that each synergy is responsible for. Finally, using the physical interpretation of each synergy, along with the discrepancies observed between group, this study determined the possible malfunctioning sub-tasks in severe slippers which cause persons to experience more severe slips rather than mild slips. Also, while there were no differences in age, height, and mass observed between the two severity groups, there were several significant differences in the slip responses (reflected as differences in muscle synergies) and motor control of mild and severe slippers. Consequently, these points together suggest that the slip severity outcome may be associated with the slip response of the individual rather than other physical differences.

The results of this study could potentially result in development of a targeted motor-rehabilitation based on the deficient muscle synergies. Such trainings will aim at re-establishing the lost or impaired muscle synergies (and the corresponding sub-tasks). The efficacy of such a training will be tested in future studies. Synergy-based targeted motor-rehabilitation, if found effective, would be more convenient and practical, as it addresses only the lost sub-task (less complex to practice), instead of the original motor-task (more complex to practice).

## Author Contributions

MMN worked on data analysis, and writing the manuscript. KB worked on data acquisition and data analysis. PH worked on idea conception, data analysis, and writing the manuscript.

## Conflict of Interest Statement

The authors declare that the research was conducted in the absence of any commercial or financial relationships that could be construed as a potential conflict of interest.

## Abbreviations

CNS: central nervous system
FFA: foot floor angle
MG: medial gastrocnemius
MH: medial hamstring
NS: non-slipping (limb)
PHS: Peak Heel Speed
RF: rectus femoris
S: slipping (limb)
TA: tibialis anterior
